# Mechanisms of endoderm formation in a cartilaginous fish reveal ancestral and homoplastic traits in jawed vertebrates

**DOI:** 10.1242/bio.20148037

**Published:** 2014-10-31

**Authors:** Benoit G. Godard, Marion Coolen, Sophie Le Panse, Aurélie Gombault, Susana Ferreiro-Galve, Laurent Laguerre, Ronan Lagadec, Patrick Wincker, Julie Poulain, Corinne Da Silva, Shigehiro Kuraku, Wilfrid Carre, Agnès Boutet, Sylvie Mazan

**Affiliations:** 1 Sorbonne Universités, UPMC Univ Paris 06, CNRS, UMR 7150, 29688 Roscoff, France; 2 Université d'Orléans-CNRS, UMR 6218, 45070 Orléans, France; 3 Plateforme d'Imagerie, Sorbonne Universités, UPMC Univ Paris 06, CNRS, FR 2424, Station Biologique, 29688 Roscoff, France; 4 CEA-Institut de Génomique-Genoscope, 2 rue Gaston-Crémieux, 91057 Evry, France; 5 Genome Resource and Analysis Unit (GRAS), Center for Developmental Biology, RIKEN.2-2-3 Minatojima-minami, Chuo-KU, Kobe 650-0047, Japan; 6 ABiMS, Sorbonne Universités, UPMC Univ Paris 06, CNRS, FR 2424, 29688 Roscoff, France; 7 Present address: CNRS UPR 3294, Institute of Neurobiology Alfred Fessard, 91198 Gif-sur-Yvette, France.; 9 Present address: UMR 7355, Université d'Orleans-CNRS, 45071 Orléans, France.; 8 Present address: Instituto de Neurociencias, Consejo Superior de Investigaciones Científicas y Universidad Miguel Hernández, Campus San Juan de Alicante, 03550 Alicante, Spain.

**Keywords:** endoderm, telolecithal egg, chondrichthyan, Nodal signalling

## Abstract

In order to gain insight into the impact of yolk increase on endoderm development, we have analyzed the mechanisms of endoderm formation in the catshark *S. canicula*, a species exhibiting telolecithal eggs and a distinct yolk sac. We show that in this species, endoderm markers are expressed in two distinct tissues, the deep mesenchyme, a mesenchymal population of deep blastomeres lying beneath the epithelial-like superficial layer, already specified at early blastula stages, and the involuting mesendoderm layer, which appears at the blastoderm posterior margin at the onset of gastrulation. Formation of the deep mesenchyme involves cell internalizations from the superficial layer prior to gastrulation, by a movement suggestive of ingressions. These cell movements were observed not only at the posterior margin, where massive internalizations take place prior to the start of involution, but also in the center of the blastoderm, where internalizations of single cells prevail. Like the adjacent involuting mesendoderm, the posterior deep mesenchyme expresses anterior mesendoderm markers under the control of Nodal/activin signaling. Comparisons across vertebrates support the conclusion that endoderm is specified in two distinct temporal phases in the catshark as in all major osteichthyan lineages, in line with an ancient origin of a biphasic mode of endoderm specification in gnathostomes. They also highlight unexpected similarities with amniotes, such as the occurrence of cell ingressions from the superficial layer prior to gastrulation. These similarities may correspond to homoplastic traits fixed separately in amniotes and chondrichthyans and related to the increase in egg yolk mass.

## INTRODUCTION

Spectacular expansions of the egg yolk mass have taken place several times during vertebrate evolution, extreme examples of this evolutionary trend being observed in amniotes, cartilaginous fishes and myxinoids. These adaptations have gone together with transitions from holoblastic to meroblastic cleavage modes and major changes in the early embryo architecture (Arendt and Nübler-Jung, 1999). One of the most visible examples of such changes is the presence of endodermal components morphologically distinct from the embryonic gut and traditionally referred to as extraembryonic, such as the early lower layer of birds (hypoblast and endoblast), primitive endoderm of mammals or yolk syncytial layer (YSL) of teleosts. The cellular organization and mode of formation of these tissues have been extensively studied in the mouse, chick and zebrafish and they appear highly divergent between these species. For instance, in the mouse, the primitive endoderm forms a morphologically distinct polarized epithelium, which arises from the blastocyst inner cell mass by a cell sorting mechanism, shortly after blastocoel formation. It later subdivides into two components, the parietal endoderm and visceral endoderm, which contrary to the traditional view of an absolute early segregation between embryonic and extraembryonic tissues, is now known to contribute to the gut (Rossant and Tam, 2009; Kwon et al., 2008). In the chick, hypoblast formation has long been thought to involve the merging of cell clusters, originating from the early epiblast by poly-ingression, and the occurrence of single cell ingressions from the epiblast prior to gastrulation has recently been confirmed at stages preceding primitive streak formation (Stern and Downs, 2012; Voiculescu et al., 2014). Finally, the zebrafish YSL forms by a collapse of marginal blastomeres with the yolk cell cytoplasmic cortex between the 512- to 1024-cell stage (Carvalho and Heisenberg, 2010). Despite these differences in morphogenesis, the amniote hypoblast/AVE (anterior visceral endoderm) and teleost dorsal YSL share expression of signaling molecules and transcription factors known as components of AME (anterior mesendoderm) genetic programs, a similarity proposed to be related to independent recruitments in the amniote and actinopterygian lineages (Stern and Downs, 2012).

How the increase in egg yolk amount may affect early endoderm formation and patterning is poorly known outside osteichthyans (bony fishes and their descendants, including tetrapods), which comprise all established vertebrate model organisms. Cartilaginous fishes or chondrichthyans, which form one of the three major vertebrate phyla and comprise about 1100 extant species, are of interest to address this issue for two reasons. First, as the closest outgroup to osteichthyans, the other major phylum of gnathostomes (jawed vertebrates), they are essential to reconstruct gnathostome ancestral characteristics, through comparisons with other vertebrate models (Coolen et al., 2008a). Second, a lecithotrophic mode of embryonic nutrition is likely to be ancestral in chondrichthyans and most elasmobranchs develop from large telolecithal eggs, endowed with a distinct yolk sac (Blackburn, 2014). This is in particular the case of the catshark *Scyliorhinus canicula*, one of the most extensively studied representatives of chondrichthyans (Coolen et al., 2008a). This species develops from large, telolecithal eggs, which undergo a discoidal meroblastic cleavage and are laid at early stages of blastocoel formation (Ballard et al., 1993). Following egg deposition, the blastoderm consists of two cell layers, a superficial one, exhibiting an epithelial-like morphology, and an inner cell population of dispersed blastomeres, referred to as deep mesenchyme (Ballard et al., 1993; Coolen et al., 2007). This bilayered structure persists for about seven days, a period characterized morphologically by a size expansion of the blastoderm (Ballard et al., 1993). A marked change is observed at stage 11, which is considered as the start of gastrulation. At this stage, a novel cell population identified as mesendoderm, based both on molecular characterization and histology, starts to involute along the blastoderm posterior margin, adjacent to the deep mesenchyme (Ballard et al., 1993; Coolen et al., 2007). This involution movement results in the formation of a posterior overhang, which initially elongates over the yolk from anterior to posterior and is later found lining the developing archenteron (Coolen et al., 2007). Concomitantly, lateral and anterior regions of the blastoderm become thinner and spread over the yolk to later form a distinct yolk sac, connected to the embryo via a vascularized stalk. From the morphological appearance of the embryonic axis (stage 12) and until neural tube closure, the developing embryo thus appears strictly restricted to the posterior part of a growing, flattened disc (Ballard et al., 1993; Coolen et al., 2007). This posterior restriction of embryo formation is a major difference with teleosts and was previously suggested to be related to the increase in egg yolk mass and the rise of a distinct yolk sac (Arendt and Nübler-Jung, 1999). The timing of specification and mode of formation of early endodermal tissues are completely unknown in the catshark. Here, we address these processes, thus providing the first characterization of endoderm formation in a chondrichthyan. Comparisons with osteichthyan model organisms highlight characteristics likely to correspond to ancestral traits of jawed vertebrates, and homoplastic features with amniotes, possibly indicative of conserved developmental constraints.

## MATERIALS AND METHODS

### Embryo production and maintenance, staging and nomenclature

*S. canicula* eggs were produced by the Biological Marine Resources facility of Roscoff Marine Station and kept in 17°C oxygenated sea water until the desired stages were obtained. This study was performed on catshark embryos prior to formation of the nervous system and of any other organ and is therefore exempt from a special license under the terms of institutional and national regulations. Embryos were staged after Ballard et al. (Ballard et al., 1993) and a description of the stages studied is provided in supplementary material Movies 1 and 2. Stage 11 is considered as the start of gastrulation, based on two criteria (1) the appearance of a distinct mesendoderm layer (Ballard et al., 1993) and (2) the onset of *Brachyury* expression (Coolen et al., 2007). Prior to stage 11, the anterior to posterior polarity of the blastoderm refers to the orientation of the future elongating embryonic axis, and corresponds to the ventral to dorsal, or ab-organizer to organizer polarity of amphibians and teleosts.

### Probe isolation and characterization

The *S. canicula Dkk1* probe was amplified by degenerate RT-PCR from stage 9–15 cDNA by a nested PCR, successively using the following pairs of primers: 5′-GAYGCNATGTGYTGYCC and 3′-ATYTTRCTCCARAARTG, respectively encoding the conserved DAMCCP and HFWSKI amino acid motifs of the Dkk1 peptide of other vertebrates, and 5′-GCNATGTGYTGYCCNGG and 3′-ARRCACATRTCNCCYTC, respectively encoding the conserved motifs AMCCPG and EGDMCL. The amplified cDNA fragments were subcloned in the pGEM-T easy vector and sequenced. *ScSox17*, *ScHex*, *ScLeftyB*, *ScFgf17*, *ScShh* and *ScChd* probes were obtained from a large-scale cDNA sequencing project described (Coolen et al., 2007). Novel sequences were included in molecular phylogenetic trees to confirm their identity (supplementary material Fig. S1). *ScT*, *ScOtx5*, *ScGata6* and *ScLim1* probes were reported in previous studies (Coolen et al., 2007; Plouhinec et al., 2005; Sauka-Spengler et al., 2003).

### In situ hybridization and histological analyses

Whole-mount in situ hybridizations were conducted using standard protocols adapted to the catshark and followed by embryo embedding in paraffin and sectioning, as described previously (Derobert et al., 2002). For semi-thin sections, embryos were fixed in 4% glutaraldehyde, 0.25 M sucrose in 0.2 M cacodylate buffer pH 7.4, post-fixed in 1% OsO4 and embedded in Epon. 0.5 µm sections were cut and stained with toluidine blue.

### DiI cell labeling

Stage 8 to 10 embryos were removed from the shell and transferred to 0.45 µm filtered sea water. CellTracker CM-DiI (Invitrogen) was diluted (1/10) in 0.3M sucrose from a 5 mg/ml stock solution in ethanol and applied to embryo territories by ejection from a capillary tube. A control was also performed after one hour of culture, in order to check the absence of internal labeling due to tissue disruption related to the process of dye application. Labeled embryos were cultured in filtered sea water for 24 hours, prior to fixation, paraffin embedding and sectioning (12 µm). Sections were stained with DAPI, mounted and photographed using a Leica SP5 confocal microscope. The presence or absence of labeled cells was assessed in the deep mesenchyme or involuted mesendoderm, taking into account heavily labeled cells.

### Pharmacological treatments

Pharmacological treatments were conducted by in ovo injection of 200 µl of a 500 µM dilution of the Alk4/5/7 inhibitor SB-505124 in 0.01% DMSO in stage 8/9 catshark embryos. This solution was replaced by the same volume of 0.01% DMSO in control embryos. Following injections, eggs were maintained for 3 days in oxygenated sea water at 17°C, with viabilities higher than 90%. They were then dissected, fixed in PFA 4%, dehydrated and stored at −20°C in methanol 100% prior to in situ hybridization.

## RESULTS

### Two distinct phases of *ScSox17*, *ScGata6* and *ScHex* expressions in the early catshark embryo

**Fig. 1.**
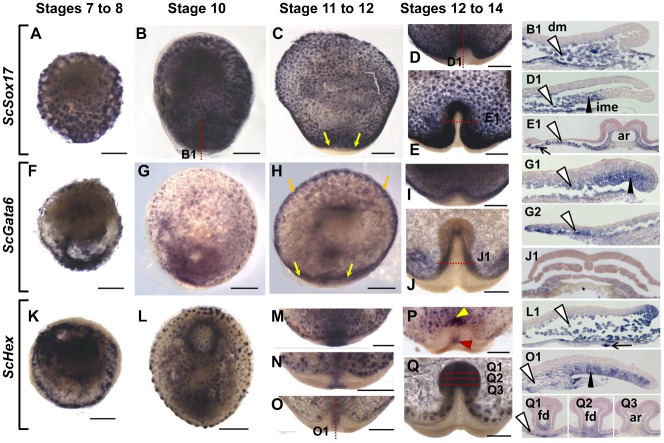
Characterization of endodermal tissues in the catshark: *ScGata6*, *ScSox17* and *ScHex* expressions from early blastula to axis elongation stages. Dorsal views of *S. canicula* embryos following whole-mount in situ hybridizations with *ScSox17*, *ScGata6* and *ScHex* probes respectively. Embryos stages are as follows: (A,F,K) stages 7–8. (B,G,L) stage 10 embryos; (C,H) stage 11 embryos; (M–O) magnifications of the posterior margin of stage 11, 12− and 12 embryos; (D,I,P) magnifications of the posterior margin of stage 12 (D,I) to 12+ (P) embryos; (E,J,Q) magnifications of the elongating axis of stage 14 embryos. Sections of the embryos shown in panels B, D, E, G, J, O and Q are shown on the right in panels B1, D1, E1, J1, O1 and Q1–3 as indicated on whole-mount views, with section planes shown as red dotted lines. (G1,G2) Sections of the posterior and anterior margin of a stage 10+ embryo hybridized with *ScGata6*, (L1) section of the posterior margin of a stage 10 embryo hybridized with *ScHex*. White arrowheads point to labeled cells in the deep mesenchyme, black arrowheads point to the labeled part of the involuting mesendoderm, thin arrows point to labeled yolk syncytial nuclei. Yellow arrows in panels C and H delimit a midline territory of the involuting mesendoderm expressing *ScGata6* but not *ScSox17*. Orange arrows in panel H point to the *ScGata6* positive, *ScSox17* negative anterior and lateral margins of the blastoderm at stage 11, which express markers of lateral mesoderm. The red and yellow arrowheads in panel P show *ScHex* signals respectively in the presumptive prechordal mesendoderm and in the anteriormost region of the involuting mesendoderm adjacent to the deep mesenchyme. Abbreviations used: ar, archenteron; ime, involuting mesendoderm; dm, deep mesenchyme; fd, foregut diverticulum; lm, mesoderm of lateral identity. Scale bars: 500 µm.

In order to unambiguously identify endodermal cell populations in the catshark, we analyzed expression of homologues of three genes known to be expressed in extraembryonic endoderm in amniotes and additional mesendoderm territories, *ScSox17*, *ScGata6*, and *ScHex*. Expression was observed at the earliest blastula stages studied (stages 4–6), less than 48 hours following egg deposition (supplementary material Fig. S2A). At stages 7 to 10, all three were expressed in the deep mesenchyme in contact with the yolk, as well as in and around large, subjacent yolk syncytial nuclei (Fig. 1A,B,F,G,K,L; sections in Fig. 1B1,G1,G2,L1). The deep mesenchyme persists in blastoderm territories, which lie adjacent to the elevating embryonic axis and spread anteriorly and laterally over the yolk at subsequent stages (supplementary material Movies 1 and 2; Fig. S2F,G). Expressions of *ScSox17*, *ScGata6*, and *ScHex* in this tissue were maintained from stage 11 to at least stage 14 (Fig. 1C–E,H–J,M–Q; sections in Fig. 1D1,E1,J1,O1,Q1–Q3; see also supplementary material Fig. S2B,D–G). Starting from stage 11, expression of all three genes was also observed in different territories of the involuting AME layer. *ScSox17* transcripts accumulated in the involuted mesendoderm along a 60° crescent of the posterior margin (Fig. 1C,D,D1; supplementary material Fig. S2D). *ScGata6* territory largely overlapped with *ScSox17*, the signal extending further laterally in marginal territories known to express lateral mesoderm markers (Fig. 1H,I; supplementary material Fig. S2E). *ScHex* expression appeared in the involuting AME at stage 11, initially confined to the midline of the posterior margin (Fig. 1M) and progressively displaced to the anterior aspect of the involuting layer and adjacent deep mesenchyme as involution proceeded (Fig. 1N–P,O1). An additional signal was also transiently observed more posteriorly, in the prospective prechordal mesendoderm (Fig. 1P; supplementary material Fig. S2C). At stages 13–14, expression of all three genes was maintained in the ventral and lateral parts of the forming foregut of the embryonic axis as it elevates (Fig. 1E,J,Q and corresponding sections Fig. 1E1,J1,Q1–Q3).

### Deep mesenchyme formation in the catshark

**Fig. 2.**
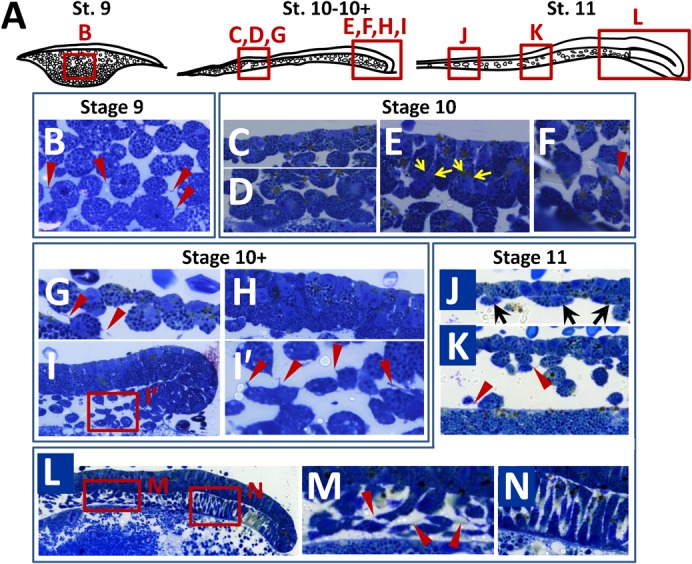
Histological analysis of deep mesenchyme formation in *S. canicula* from mid-blastula to early gastrula stages. (A) Schemes of mid-sagittal sections of catshark embryos at the stages indicated. The cellular organization of the territories boxed in red is shown in panels B–N. (B–N) Semi-thin (0.5 µm) sections of the territories boxed in panel A, following toluidine Blue staining. (B) Stage 9: cellular organization of the deep mesenchyme. (C–F) Stage 10: (C) cuboidal epithelial-like structure of the superficial cell layer and (D) sub-jacent deep mesenchyme in the anterior region of the blastoderm; (E) columnar epithelial-like structure of the superficial cell layer and (F) sub-jacent deep mesenchyme in the posterior region of the blastoderm. (G–I′) Stage 10+: (G) cuboidal epithelial-like structure of the superficial cell layer and sub-jacent deep mesenchyme in the anterior region of the blastoderm; (I) general structure of the posterior blastoderm margin and (I′) higher magnification of the deep mesenchyme in the territory boxed in panel I, adjacent to the posterior margin; (H) columnar epithelial-like structure of the superficial cell layer. (J–N) Stage 11: (J,K) cuboidal epithelial-like structure of the superficial cell layer and sub-jacent deep mesenchyme in the anterior margin (J) and a more central region of the blastoderm (K); (L) general structure of the posterior involuting blastoderm margin and adjacent deep mesenchyme; (M,N) higher magnifications of panel L showing elongated cells in the deep mesenchyme adjacent to the involuting AME (M) and the cellular organization of the involuting cell layer in its anterior-most aspect (N). Red arrowheads in panels B, G, I′, K and M point to cell protrusions in the deep mesenchyme. Black arrows in panel J show flattened deep cells appearing at this stage close to the anterior margin. Yellow arrows in panel E point to cells showing apical constrictions suggestive of internalizations from the superficial layer.

**Fig. 3.**
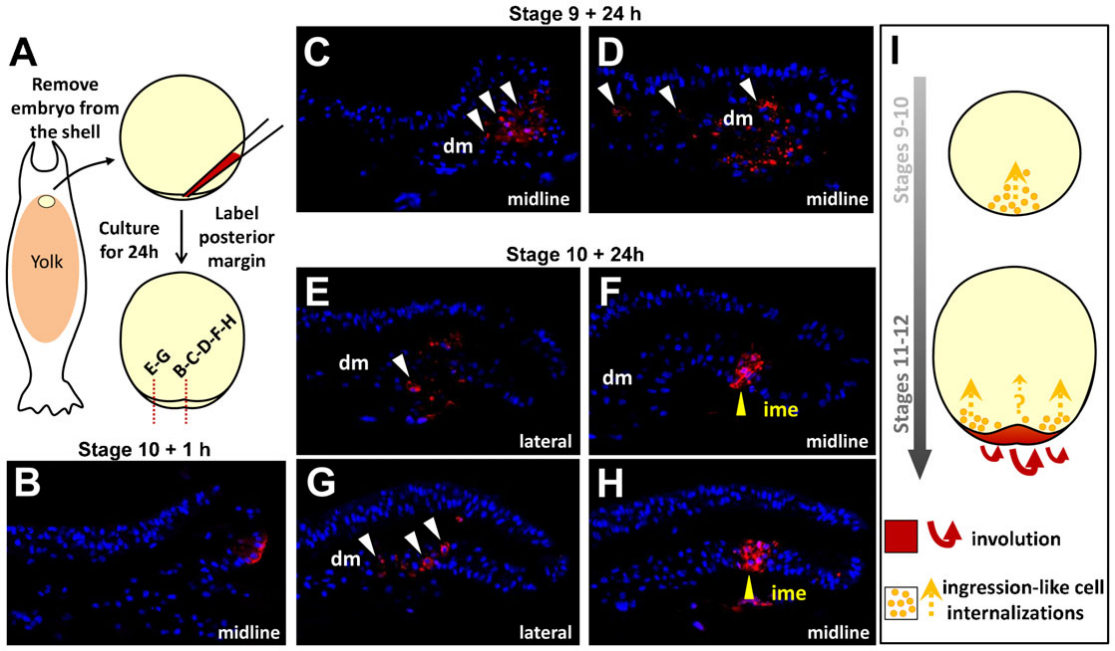
Temporal regulation of cell behaviors at the posterior margin of *S. canicula* embryos from late blastula to early gastrula stages. (A) Scheme showing the experimental procedure used, and the plane and location of the sections shown in panels B–H (red dotted lines). (B–H) DAPI staining (blue) and DiI fluorescence detection (red) on sections of embryos labeled as in panel A. (B) Example of a control stage 10 labeled embryo, cultured for one hour following DiI application. (C,D) Mid-sagittal sections of two embryos labeled in the midline at stage 9 and cultured for 24 hours after DiI application. (E,F) Respectively para-sagittal and mid-sagittal sections of an embryo labeled at lateral and medial levels at stage 10 and cultured for 24 hours after DiI application. Same in panels G,H, with DiI application at stage 10+. White and yellow arrowheads point to DiI labeled cells in the deep mesenchyme or the involuting mesendoderm respectively. Panel I schematizes the types of movements observed for cells derived from the posterior margin depending on both stage and location along the margin (see Results). Whether internalizations by an ingression-like process take place in early gastrulae at the transition zone between the involuting mesendoderm and deep mesenchyme (i.e. the anterior-most aspect of the involuting layer) could not be addressed, due to the inaccessibility of this territory and limitations in embryo culture times (orange question mark). Same abbreviations as in Fig. 1.

**Fig. 4.**
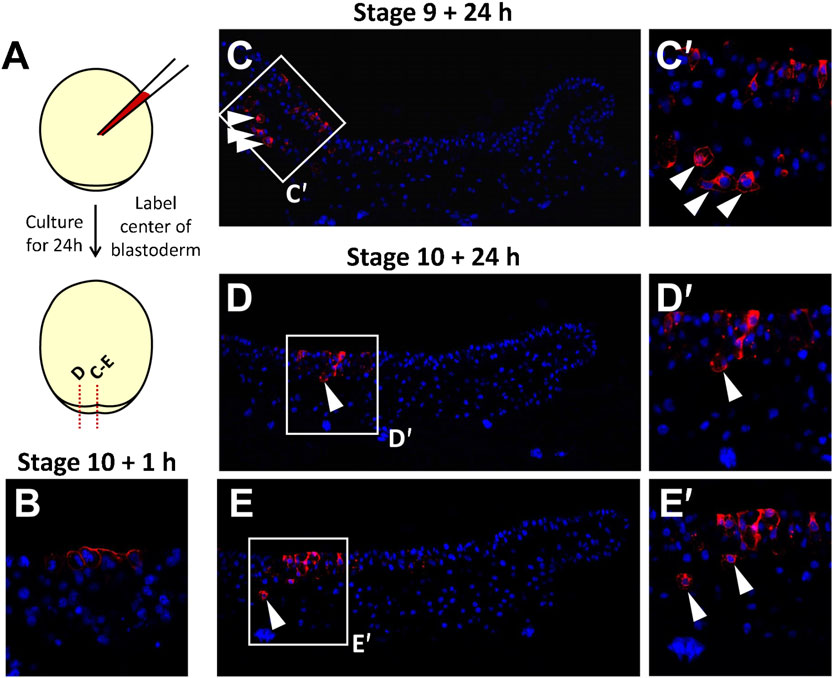
Cell internalizations from the superficial layer at the center of the blastoderm from stages 9 to 10 in *S. canicula*. (A) Scheme showing the experimental procedure and plane and location of the sections shown in panels C–E (red dotted lines). (B–E) DAPI staining (blue) and DiI fluorescence detection (red) on sections of embryos labeled as in panel A. (B) Example of a control stage 10 labeled embryo, cultured for one hour following DiI application. (C) Mid-sagittal section of an embryo labeled in the center of the blastoderm at stage 9 and cultured for 24 hours after DiI application. (C′) Higher magnification of the territory boxed in panel C showing labeled internalized cells. (D,E) Respectively para-sagittal and mid-sagittal sections of an embryo labeled in the center of the blastoderm at stage 10 and cultured for 24 hours after DiI application. (D′,E′) Higher magnification of the territories boxed in panels D and E showing labeled internalized cells. White arrowheads point to DiI labeled cells in the deep mesenchyme.

In order to gain insight into the mode of formation of the deep mesenchyme, we next conducted a histological description, based on analysis of semi-thin sections from stage 9 to stage 11. This analysis highlighted the presence of several populations of inner cells, differing by their morphology. At stage 9, all cells appeared round shaped, with thin randomly oriented protrusions (Fig. 2A,B). At stages 10–10+, this cell morphology persisted in the anterior part of the blastoderm, lying underneath the superficial layer (Fig. 2C,D,G; see also Fig. 2K at stage 11) but cells showing an altered morphology appeared in the posterior part of the blastoderm, close to the posterior margin (Fig. 2F,I,I′). At these stages, the superficial layer of the posterior part of the blastoderm displayed a columnar morphology (Fig. 2E,H), as previously reported (Coolen et al., 2007) and cells exhibiting apical constrictions suggestive of internalizations could frequently be observed (Fig. 2E). At stage 11, two novel cell morphologies could be observed in the deep mesenchyme, (i) flattened cells lying beneath the superficial layer at the extreme anterior part of the blastoderm (Fig. 2J) and (ii) elongated cells located adjacent to, and anterior to the involuting layer, close to the yolk cell (Fig. 2L,M). The latter exhibited protrusions oriented along the AP (antero-posterior) axis, perpendicular to those of the adjacent AME involuting layer (compare Fig. 2M,N). These cell morphologies suggest that the formation of the deep mesenchyme may involve single cell internalizations and migrations from the superficial layer and posterior margin. To directly address this possibility, we used DiI cell labeling to track cells originating from these locations from mid-blastula to early gastrula stages (Fig. 3, Fig. 4). After one hour of culture following local applications of the DiI solution either at the posterior margin or at the center of the blastoderm (Fig. 3A, Fig. 4A; supplementary material Tables S1 and S2), labeled cells formed a single, superficial territory comprising 5 to 20 fluorescent cells (Fig. 3B, Fig. 4B) and were never observed either in the deep mesenchyme or involuting mesendoderm (supplementary material Tables S1 and S2). Labeled embryos were then cultured for 24 hours after DiI application and the location of fluorescent cells was examined on histological sections. In the youngest embryos injected at the posterior margin (stage 8–9; stage 10 after culture; n = 4), all fluorescent cells were found internalized as a cluster of mesenchymal cells, close to the site of injection (Fig. 3C,D; supplementary material Table S1). A marked change in the organization of fluorescent cells was observed when DiI was applied at the posterior margin at subsequent stages (stages 10/11). In these embryos, labeled cells were found displaced within the involuting mesendoderm layer as a highly coherent group but never observed in the deep mesenchyme (Fig. 3F,H; supplementary material Table S1; n = 4). DiI application at the center of the blastoderm at stages 8 (mid-blastula) to 10 (late blastula) also led to the presence of labeled cells in the deep mesenchyme in all embryos studied after culture (Fig. 4C–E,C′–E′; supplementary material Table S2; n = 6). In this case however, the superficial layer remained heavily labeled at the site of dye application, which was not observed when the dye was applied at the level of the posterior margin. Of note is that during early gastrulation, application of the dye at lateral levels of the margin resulted in an organization of fluorescent daughter cells similar to the one observed at the posterior midline at earlier stages (Fig. 3E,G). Thus, as development proceeds, cell internalizations progress laterally along the posterior margin, following the same succession of distinct cell behaviors as observed in the midline (Fig. 3I).

### The posterior deep mesenchyme and AME share expression of the same signaling molecules

**Fig. 5.**
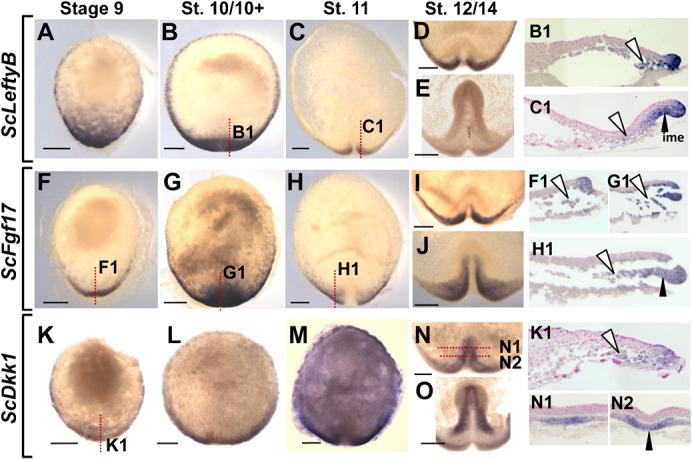
Expression of signaling molecules at the posterior margin and in the forming embryonic axis of catshark embryos from stages 9 to 14. Dorsal views of embryos following whole-mount in situ hybridization with *ScLeftyB*, *ScFgf17* and *ScDkk1* probes respectively. Views in panels D, E, I, J, N and O are restricted to the posterior part of the blastoderm where elongation of the embryonic axis takes place. Stages are as follows: (A,F,K) stage 9 embryos; (B,G,L) stage 10/10+; (C,H,M) stage 11; (D,I,N) stage 12; (E,J,O) stage 14. Sections of the embryos photographed in panels B, C, F, G, H, K and N are shown as indicated in panels B1, C1, F1, G1, H1, K1, N1 and N2, with the plane and level of section indicated by a red dotted line. White arrowheads point to labeled cells in the deep mesenchyme, black arrowheads point to the labeled part of the involuting mesendoderm. Same abbreviations as in Fig. 1. Scale bars: 500 µm.

**Fig. 6.**
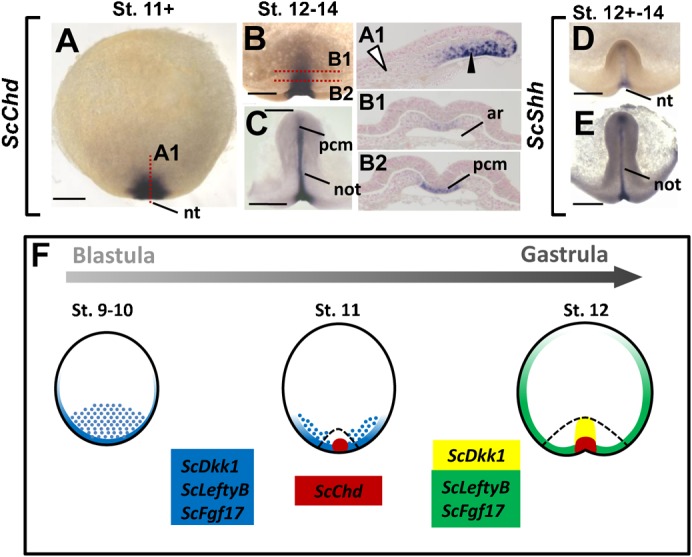
Expression of *ScChd* and *ScShh* at stages 11–14 and comparison with *ScLeftyB*, *ScFgf17* and *ScDkk1* patterns. (A–C) Dorsal views of embryos following whole-mount in situ hybridization with an *ScChd* probe. Views in panels B and C are restricted to the posterior part of the blastoderm where elongation of the embryonic axis takes place. Stages are as follows: (A) stage 11+; (B) stage 12; (C) stage 14. Sections of the embryos photographed in panels A and B are shown in panels A1, B1 and B2, with the plane and level of section indicated by a red dotted line. White arrowheads point to the *ScChd* negative deep mesenchyme, black arrowheads point to the labeled part of the involuting mesendoderm. (D,E) Dorsal views of the posterior margin following whole-mount in situ hybridizations with *ScShh*. (F) Scheme summarizing the expression patterns observed in endodermal tissues in the catshark. Dotted areas indicate signals in the deep mesenchyme, uniformly colored areas indicate signals in the involuting mesendoderm and at the margin. A dotted black line delimits the extent of the involuting mesendoderm. The combination of genes expressed in each territory is indicated by the color code shown on the left of the scheme. Abbreviations used: ar, archenteron; nt, notochordal triangle; not, notochord; pcm, prechordal mesendoderm. Scale bars: 500 µm.

In line with their roles in embryo patterning or germ layer specification, the amniote AVE or hypoblast are a source of secreted signals, such as Fgf8 or the Nodal and Wnt antagonists Lefty and Dkk1. These signals differ from those later secreted by the organizer, which expresses the BMP antagonist Chordin. In order to address the signaling properties of the deep mesenchyme, we analyzed expression of catshark orthologues of *Lefty*, *Dkk1* and of *Fgf17*, a member of the *Fgf8/Fgf17/Fgf18* class, from early blastula stages to 14. No expression of a *Lefty* orthologue (referred to hereafter as *ScLeftyA*) was previously detected in this tissue (Coolen et al., 2007). We reassessed this conclusion by analyzing a second *Lefty* orthologue, termed *ScLeftyB*, isolated from additional EST sequencing (supplementary material Fig. S1). *ScLeftyB* and *ScFgf17* expressions were already detectable at the posterior margin less than 48 hours after egg deposition (stage 4 to 6, supplementary material Fig. S2A). From stages 9 to 11, they showed expression characteristics very similar to *ScDkk1*. At stages 9–10+, all three shared a prominent positive territory at the posterior margin and the adjacent deep mesenchyme (Fig. 5A,B,F,G,K,L and corresponding sections 5B1,F1,G1,K1). At stage 11, the signal also disappeared for all three genes from the medial-most part of the margin, persisting more laterally (Fig. 5C,H,M, sections in Fig. 5C1,H1). Their territories withdrew from the deep mesenchyme and segregated at subsequent stages. At stages 12–13, expression of all three genes persisted in the mesendoderm at the posterior arms level (Fig. 5D,I,N) and in the case of *ScDkk1*, in its anteriormost component at the midline level (Fig. 5N,N1,N2). These broad expression characteristics were maintained in the elongating embryonic axis except for *ScLeftyB*, restricted at stage 14 to a small midline territory of the forming trunk (Fig. 5E,J,O). In order to compare these profiles with those of an organizer specific marker, we next analyzed *ScChd*, the catshark orthologue of *Chordin*. *ScChd* expression remained undetectable at stage 10 but a strong signal was observed at stage 11 in a medial domain of the involuting posterior margin (Fig. 6A,A1), where a morphological structure referred to as the notochordal triangle later becomes visible (Sauka-Spengler et al., 2003). At later stages, expression was restricted to this structure, excluding the *ScT* positive posterior arms (Fig. 6B,C). *ScShh*, the catshark orthologue of *Shh*, was also expressed in the notochordal triangle starting from stage 12 (Fig. 6D,E). At later stages, as in other vertebrates, *ScChd* and *ScShh* were expressed in the forming notochord, visible in the catshark from stage 13 (Fig. 6C,E). Unlike *ScLeftyB*, *ScFgf17*, *ScDkk1* and *ScLim1* (Coolen et al., 2007), *ScChd* expression was never observed in the deep mesenchyme and it never reached the anteriormost region of the involuting layer (Fig. 6A1; summary in Fig. 6F). At later stages, it was also excluded from the foregut diverticulum, the signal intensity exhibiting a gradual decrease from chordal to prechordal levels of the axial mesendoderm (compare Fig. 6B1 and Fig. 6B2).

### Nodal/activin signaling is essential for the regionalization of the deep mesenchyme and the specification of the posterior margin

**Fig. 7.**
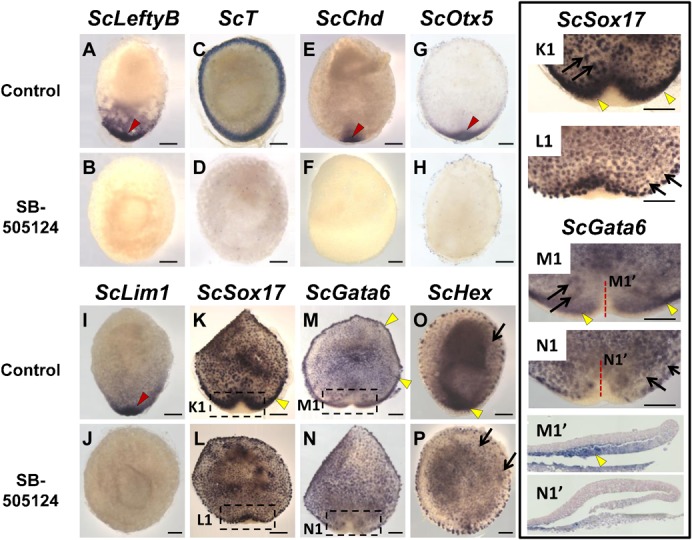
Effect of the Nodal/activin antagonist SB-505124 on *S. canicula* development at late blastula to early gastrula stages. Dorsal views of catshark embryos following whole-mount in situ hybridizations with the following probes: *ScLeftyB* (A,B), *ScT* (C,D), *ScChd* (E,F), *ScOtx5* (G,H), *ScLim1* (I,J), *ScSox17* (K,L), *ScGata6* (M,N), *ScHex* (O,P). (K1,L1,M1,N1) Higher magnifications of panels K, L, M, N at the level of boxed territories. (A,C,E,G,I,K,M,O) Control embryos. (B,D,F,H,J,L,N,P) SB-505124 treated embryos. All treated embryos shown are class 1 embryos, except those shown in panels L and N, which show examples of class 2 embryos. Control embryos are stage 11 embryos, except those shown in panels K and M, which are stage 12 embryos. (M1′,N1′) Mid-sagittal sections of the posterior margin of control and SB-505124 treated class 2 embryos respectively, following hybridization with a *ScGata6* probe. Red arrowheads point to *ScLeftyB*, *ScChd*, *ScOtx5* and *ScLim1* signals at the posterior margin and adjacent inner cell populations (involuting mesendoderm and/or deep mesenchyme), visible in the controls but absent in treated embryos. Yellow arrowheads point to the second phase of *ScSox17*, *ScGata6*, and *ScHex*, observed in control embryos but undetectable following SB-505124 treatment. Thin arrows point to signals in the deep mesenchyme and syncytial nuclei, which are maintained in treated embryos. Red dotted lines in panels M1 and N1 indicate the section planes shown in panels M1′ and N1′. Scale bars: 500 µm.

Nodal/activin signaling is essential for the specification of mesoderm and gut endoderm in all vertebrates studied and also required for AVE specification in the mouse (Mesnard et al., 2006). In order to analyze the role of Nodal/activin signaling in endoderm development in the catshark, we conducted an in ovo pharmacological approach, using SB-505124, a selective inhibitor of activin Alk4/5/7 receptors. The drug or control DMSO was injected inside the eggshell at stages 8 to 9, reached about 5 days following egg deposition. Eggs were then maintained for three days in 17°C oxygenated sea water prior to embryo fixation and dissection. DMSO injected control embryos appeared normal and their stages ranged between 11 (n = 21) and 12 (n = 7), as expected for uninjected embryos at this temperature. SB-505124 treated embryos could be classified into two classes based on their general morphology (supplementary material Table S3). The majority, referred to as class 1 embryos, appeared as flattened blastodiscs, without evidence of posterior fold formation (Fig. 7B,D,F,H,J,P). A minority of treated embryos, referred to as class 2 embryos, showed similarities to stage 12 embryos, in that they exhibited distinct posterior arms on each side of the forming embryonic axis (Fig. 7L,N). In order to assess the loss of Nodal/activin signaling in the experimental conditions tested, we first focused on expression of the feedback antagonist *ScLeftyB* in SB-505124-treated and control embryos (supplementary material Table S3; Fig. 7A,B). While present at the posterior margin in all control embryos tested (n = 3, Fig. 7A), *ScLeftyB* expression remained undetectable in all treated embryos (class 1, n = 3, Fig. 7B), in line with a loss of Nodal/activin signaling. We next analyzed expression of the general mesoderm marker *ScT* (Fig. 7C,D) and of the notochordal triangle marker *ScChd* (Fig. 7E,F). In both cases, control embryos exhibited the expected signals (n = 5 for *ScT*, n = 2 for *ScChd*) around the whole margin (*ScT*, Fig. 7C) or in the involuting axial mesendoderm (*ScChd*, Fig. 7E). In contrast, *ScT* and *ScChd* expressions were abolished in all treated embryos analyzed (n = 4 and n = 3 respectively, Fig. 7D,F). Similarly, *ScOtx5* and *ScLim1* signals were observed, in the involuting mesendoderm and adjacent deep mesenchyme of control embryos (n = 4 for *ScOtx5*, same for *ScLim*; Fig. 7G,I), but lost in all treated embryos (n = 2 and n = 4 for *ScOtx5* and *ScLim1* respectively; Fig. 7H,J). Finally, we analyzed the effect of the drug on three genes characterized by an early expression in the deep mesenchyme and syncytial nuclei, and a later expression phase at different levels of the involuting margin as shown above, *ScSox17*, *ScGata6* and *ScHex* (Fig. 7K–P). In all cases (total of 11 embryos tested), the signal in the deep mesenchyme and yolk syncytial nuclei was maintained in control and SB-505124 treated embryos (compare Fig. 7K and Fig. 7L, Fig. 7M and Fig. 7N, Fig. 7O and Fig. 7P; see also supplementary material Fig. S3A,B). In contrast, no marginal expression was observed in any of the treated embryos (n = 13, Fig. 7L,N,P), while it was present in control embryos (Fig. 7K,M,O; compare Fig. 7K1 and Fig. 7L1, Fig. 7M1 and Fig. 7N1, Fig. 7M1′ and Fig. 7N1′; see also supplementary material Fig. S3C1,C2,D1,D2). Histological sections showed that the deep mesenchyme was maintained in class 1 and class 2 embryos (supplementary material Fig. S3). An inner layer was present at the posterior margin of class 2 embryos but it appeared less expanded and thinner than in control embryos (compare Fig. 7M1′ and Fig. 7N1′).

## DISCUSSION

### Endoderm is specified in two phases in the catshark as in osteichthyans

Analysis of *Hex*, *Gata6* and *Sox17* orthologues in the catshark show that not only the anteriormost part of the involuting layer but also the deep mesenchyme is endowed with an endodermal identity. The timing of their specification appears as a major difference between these two tissues, since the latter already expresses *Sox17*, *Hex* and *Gata6* at the earliest stages accessible, which shortly follow blastocoel formation and precede gastrulation by more than seven days (Ballard et al., 1993; Coolen et al., 2007; Sauka-Spengler et al., 2003). As previously noted (Godard and Mazan, 2013), two phases of endoderm specification are most obvious in mammals, birds and teleosts, all endowed with distinct extraembryonic tissues, but have also been reported in some amphibians exhibiting no evidence for two morphologically distinct endoderm components, including xenopus. While a key role of Nodal in mesendoderm formation has been demonstrated in amniotes, amphibians and teleosts (Conlon et al., 1994; Erter et al., 1998; Feldman et al., 1998; Schier, 2009; Schier and Shen, 2000; Steiner et al., 2006), the mechanisms controlling the earliest phase of endoderm specification appear to vary extensively across vertebrates. In the catshark, formation of the involuting layer and expression of all mesoderm and mesendoderm markers were abolished following SB-505124 treatments, in line with a conservation of the role of Nodal/activin signaling in mesendoderm specification. In contrast, no evidence for a loss of the endodermal identity was observed in the deep mesenchyme following abrogation of Nodal signaling activity. This observation cannot rule out a role of Nodal signaling in the initial steps of deep mesenchyme specification, as *ScSox17*, *ScHex* and *ScGata6* expressions were already established at the time of egg laying, making it difficult to assess the effect of the drug prior to the onset of their expression. However, it argues against a major role of the pathway in the maintenance of the deep mesenchyme endodermal identity. This conclusion is also supported by the localized deep mesenchyme expression of the catshark orthologue of Lefty, a feedback antagonist of Nodal signaling (Chen and Schier, 2002; Juan and Hamada, 2001; Meno et al., 1999), which suggests a posterior restriction of Nodal signaling activity at all stages studied. From an evolutionary standpoint, the reiteration of a biphasic mode of endoderm specification now found in chondrichthyans as in all major osteichthyan lineages supports the hypothesis that it may be an ancestral characteristic of jawed vertebrates (Godard and Mazan, 2013). However, in the absence of mechanistic arguments, it remains difficult to formally exclude independent rises of the earliest specification event in the different vertebrate phyla. Finally it should be noted that a partitioning of nutritive tissues into a vegetal mass without contribution to the gut and an embryonic component derived from the blastopore margin has also been proposed in the lamprey (Takeuchi et al., 2009). However, in this species, this distinction primarily relied on the absence of endoderm marker expression in the vegetal mass, a criterion, which argues against homology with the early specified tissue identified in the catshark.

### Cell internalizations suggestive of ingression movements precede gastrulation in the catshark

**Fig. 8.**
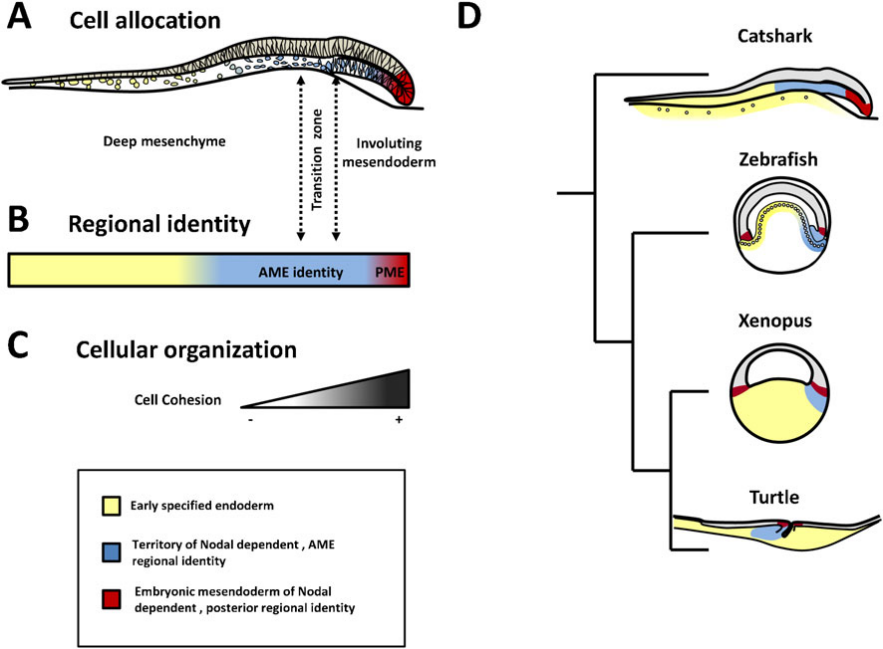
Regional pattern and cellular phenotypes in the catshark deep mesenchyme and involuting mesendoderm and comparison with other vertebrates at early gastrula stage. (A) Scheme of a mid-sagittal section of a stage 11 catshark embryo, showing the relative extent of the deep mesenchyme, involuting mesendoderm layer, and transition zone between these territories. (B) Cell regional identities in the deep mesenchyme and adjacent mesendoderm with the following color code: yellow, endoderm territory co-expressing *ScSox17*, *ScGata6* and *ScHex* but negative for anterior regional markers; blue, territory of AME identity, spanning the transition between the deep mesenchyme at the posterior side of the blastoderm and anterior mesendoderm; red, territory of posterior mesendoderm identity negative for anterior regional markers, positive for *ScChd* and *ScT*. (C) Decreased cell cohesion observed from the posterior margin to deep mesenchyme, with a shift in cell orientation at the transition zone. Abbreviations used: AME, anterior mesendoderm; PME posterior mesendoderm. (D) Comparisons of the endoderm regional pattern between the catshark, zebrafish, xenopus and turtle at the onset of gastrulation. The color code is as follows: yellow for territories exhibiting an endodermal identity, but express neither AME markers, nor *Brachyury*, blue for endoderm of anterior regional identity, red for mesendoderm of posterior regional identity.

We had previously observed that the physical continuity between the posterior deep mesenchyme and the adjacent AME goes together with a molecular continuity, both expressing not only endoderm but also AME regional markers, such as *Lim1* or *Gsc* (Coolen et al., 2007). This study extends this conclusion to signaling molecules such as the catshark orthologues of *Lefty*, *Fgf17*, a member of the Fgf8/17/18 family, or *Dkk1*, known to be specifically expressed in AME as well as in endodermal extraembryonic components of amniotes and teleosts (Stern and Downs, 2012). We further show that this regional identity is lost following abrogation of Nodal/activin signaling, which as reviewed previously (Godard and Mazan, 2013) is reminiscent of the molecular phenotype observed in the mouse embryonic visceral endoderm (Mesnard et al., 2006). Finally, we find that massive cell internalizations from the posterior margin take place prior to gastrulation and contribute to posterior deep mesenchyme formation. The posterior deep mesenchyme and involuting mesendoderm thus lie adjacent to each other, exhibit the same, Nodal-dependent, AME regional identity (Fig. 8A,B), and are also related by their embryonic origin. However, the cell internalizations from the posterior margin, which contribute to their formation, differ by their timing and the cell movements involved. The low cohesion of cells internalized at the posterior margin from stages 8 to 10, prior to the appearance of a bilayered overhang, contrasts with the tightly clustered cell organization observed at later stages, and suggests that massive ingression-like movements precede involution at the posterior margin (Fig. 8C). In addition to these cell movements taking place at the posterior margin prior to gastrulation, we also obtained evidence for cell internalizations in the center of the blastoderm. These internalizations differed from the former in that a prominent labeling persisted in the superficial cell layer following DiI application and embryo culture, suggesting that they may only concern small clusters of cells or individuals cells, as suggested by histological analyses. Taken together, these data provide evidence that early development in the catshark involves cell internalizations from the superficial layer, taking place prior to gastrulation. These movements and the shift in cell cohesion observed at the posterior margin at gastrulation are likely to involve a highly dynamic regulation of cell properties such as adhesiveness, shape, polarity and motility but the underlying mechanisms remain completely unknown. In line with the conservation of its role in mesendoderm formation across jawed vertebrates, Nodal signaling appeared essential for the formation of the involuting layer. Together with the major Lefty expression observed at the posterior margin since early blastula stages, the absence of a posterior thickening in class 1 SB-505124 treated embryos suggests that it may also control the earlier ingression-like cell movements taking place at the posterior margin. However, we could not directly assess this possibility due to the impossibility to conduct in ovo injections and DiI labeling concomitantly. Other candidate mechanisms include FGF or BMP, respectively known to regulate epithelium–mesenchyme transitions in the context of the primitive streak and the mode of migration of lateral mesoderm in the zebrafish, or Wnt-PCP and Sphingosine-1-phosphate signaling, which control the choice between individual and collective cell migrations in the zebrafish prechordal mesendoderm (Ciruna et al., 1997; Hardy et al., 2011; Kai et al., 2008; Luxardi et al., 2010; von der Hardt et al., 2007). More detailed analyses of cellular phenotypes coupled with pharmacological treatments directed against these pathways will be crucial to address this point.

### Evolutionary implications: homoplastic traits between chondrichthyans and amniotes

From an evolutionary standpoint, the cell movements, shown here to occur during catshark early development, strikingly recall some aspects of amniote development. Firstly, as already noted (Godard and Mazan, 2013) and confirmed in this study, the catshark posterior deep mesenchyme and involuting AME appear to differ by their cell organization rather than their regional identity. The establishment of fate maps in the catshark is currently hampered by difficulties to maintain embryo viability during extended durations following cell marking procedures but based on its cell organization and location relative to the developing embryonic axis, the deep mesenchyme is likely to have a major contribution to extraembryonic structures, such as the yolk sac, syncytial nuclei (Léchenault and Mellinger, 1993) or stalk connecting the embryo to the yolk sac. Deep mesenchymal cells thus persist until at least somite stages in blastoderm territories spreading over the yolk, at increasing distances from the site where involution and embryo formation take place (Ballard et al., 1993; Coolen et al., 2007; this study) (supplementary material Movie 2; Fig. S2B–G). The molecular similarity between endoderm components exhibiting very different cell organizations recalls that observed in mammals between tissues traditionally considered as extraembryonic, such as the AVE, and the adjacent AME (Fig. 8D) (Stern and Downs, 2012). The parallel is most obvious with turtles, which have retained involution as the primary mode of mesendoderm internalization a feature likely to be ancestral in amniotes (Fig. 8D) (Bertocchini et al., 2013; Coolen et al., 2008b). Secondly, the catshark also exhibits unexpected similarities with amniotes in the cell movements, which take place prior to gastrulation. In the chick, a recent study has thus demonstrated that ingressions of individual cells from the epiblast layer take place prior to the onset of gastrulation and that such movements are able to promote massive cell ingressions and induction of mesoderm markers, mimicking the formation of the primitive streak in a Nodal-dependent manner (Voiculescu et al., 2014). The early internalizations observed in the center of the blastoderm in the catshark are reminiscent of these cell movements, even though it remains unclear where they could similarly promote more massive internalizations at the posterior margin, a territory where Nodal signaling is likely to be active, based on Lefty expression. Such early cell movements, now found in the catshark as in the chick, are unlikely to represent a gnathostome ancestral characteristic since they have no equivalent either in amphibians or in teleosts. They are thus likely to correspond to a homoplastic feature (Wake et al., 2011), separately fixed in amniotes and chondrichthyans and possibly associated to the yolk increase, which took place in these lineages. Whether their rise may result from convergent evolution, involving different mechanisms, or parallel evolution, relying on the deployment of the same mechanisms and possibly indicative of developmental constraints, remains an open question. Deciphering the underlying mechanism in the chick and in the catshark will be crucial to address this issue.

### Conclusion

In conclusion, our data support the view that the blueprint of endoderm formation in jawed vertebrates involves a conserved biphasic mechanism, which tolerates an extensive variability in cell movements and individual behaviors. Such an uncoupling between highly conserved patterning mechanisms and more rapidly diverging morphogenetic processes also applies to mesoderm formation and may reflect a general evolutionary trend (Alev et al., 2013; Shook and Keller, 2008). They also point to unexpected similarities in cell movements preceding gastrulation with amniotes, likely to correspond to homoplastic features. Deciphering the mechanisms controlling the coordination between early patterning mechanisms, cell fate determination and cell behaviors in different vertebrate lineages will be essential to understand the molecular basis for the evolvability of endoderm morphogenesis across vertebrates. The catshark should be a valuable model to address these aspects, not only by its phylogenetic position, but also by the characteristics of its early development, which involves complex cell movements, remarkable temporal and spatial resolutions of the processes involved and allows straightforward comparisons with all major vertebrate model organisms.

## Supplementary Material

Supplementary Material
