# Supplementary material for: Mechanisms of endoderm formation in a cartilaginous fish reveal ancestral and homoplastic traits in jawed vertebrates
Source: Biol Open. 2014 Oct 31;3(11):1098–107. doi: 10.1242/bio.20148037 (PMC4232768; doi:10.1242/bio.20148037)
Supplement: Supplementary Material [file supp_3_11_1098__index.html]

Mechanisms of endoderm formation in a cartilaginous fish reveal ancestral and homoplastic traits in jawed vertebrates — Supplementary Material 

# Mechanisms of endoderm formation in a cartilaginous fish reveal ancestral and homoplastic traits in jawed vertebrates

## bio.20148037 Supplementary Material

**Files in this Data Supplement:**

- Supplementary Material - Benoit G. Godard et al. doi: 10.1242/bio.20148037
- Movie 1 - **Movie 1. Time lapse imaging of a developing catshark embryo (stages 6 to 10).** The movie shows a dorsal view of a catshark embryo from stages 6 to 10, anterior (referring to the orientation of the future embryonic axis) to the left. At early blastula stages (stages 6–7), the blastoderm is round-shaped, less than 1.5 mm diameter and surrounded by a characteristic white area. The blastocoel appears as a crescent located to the posterior side of the blastoderm at stage 6 and disappears at stage 7. Stages 8 to 9 (midblastula) are characterized by a size expansion and the partial (stage 8) to complete (stage 9) disappearance of the white area surrounding the blastoderm. Stage 10 is marked by the appearance of a thickening of the posterior side of the blastoderm. For the movie, a window was opened in the eggshell at the embryo level and photographs were taken every 15 minutes using an Olympus SZX12 stereomicroscope and a Qimaging colour 12-bit digital camera for the duration indicated in the movie.
- Movie 2 - **Movie 2. Time lapse imaging of a developing catshark embryo (stages 11 to 15).** The movie shows a dorsal view of a catshark embryon from stages 11 to 15, anterior to the left. The embryonic axis proper forms in the posterior part of the blastoderm, while anterior and lateral blastoderm territories spread over the yolk to form the yolk sac. Stage 11 is characterized by the morphological appearance of the involuting mesendoderm layer, which extends over the yolk at the posterior margin. At stage 12, the *ScT* positive (Sauka-Spengler et al., 2003), bilaterally symmetrical extensions which form on each side of the midline at the posterior margin are referred to as the posterior arms and the midline, *ScChordin* positive groove visible at this level (this study) is referred to as the notochordal triangle, following the nomenclature of Ballard et al. (Ballard et al., 1993). The anterior-most part of the embryonic axis, positive for *ScGsc* and *ScOtx1/2*, forms at this stage (Coolen et al., 2007). At this stage, trunk markers are restricted to the posterior arms (Coolen et al., 2007). The neural groove becomes visible in the anterior part of the forming embryonic axis at stage 13 while mesendoderm internalization progresses at the trunk level, as assessed by *ScT* and trunk markers expression (Sauka-Spengler et al., 2003; Coolen et al., 2007). At subsequent stages, the embryonic axis elongates from anterior to posterior with a sharp temporal regulation (Coolen et al., 2007). At stage 14, the neural plate exhibits an enlargement in the cephalic region, and the neural folds lying between the prospective epidermis and the neural plate become clearly delineated. At stage 15, the neural groove hollows and the neural folds get closer together (Coolen et al., 2007). We refer to the mesenchymal cell population in contact with the yolk as deep mesenchyme (dm) and to the involuting lower cell layer observed starting from stage 11 (onset of gastrulation) as involuting mesendoderm (ime). For the movie, a window was opened in the eggshell at the embryo level and photographs were taken every 15 minutes using an Olympus SZX12 stereomicroscope and a Qimaging colour 12-bit digital camera for the duration indicated in the movie.
