# Supplementary material for: Mechanisms of endoderm formation in a cartilaginous fish reveal ancestral and homoplastic traits in jawed vertebrates
Source: Biol Open. 2014 Oct 31;3(11):1098–107. doi: 10.1242/bio.20148037 (PMC4232768; doi:10.1242/bio.20148037)
Supplement: Supplementary Material [file supp_bio.20148037_bio.20148037-s1.pdf]

Biology Open (2014) 000, 1–10 doi:10.1242/bio.20148037

Supplementary Material

Benoit G. Godard et al. **doi: 10.1242/bio.20148037**

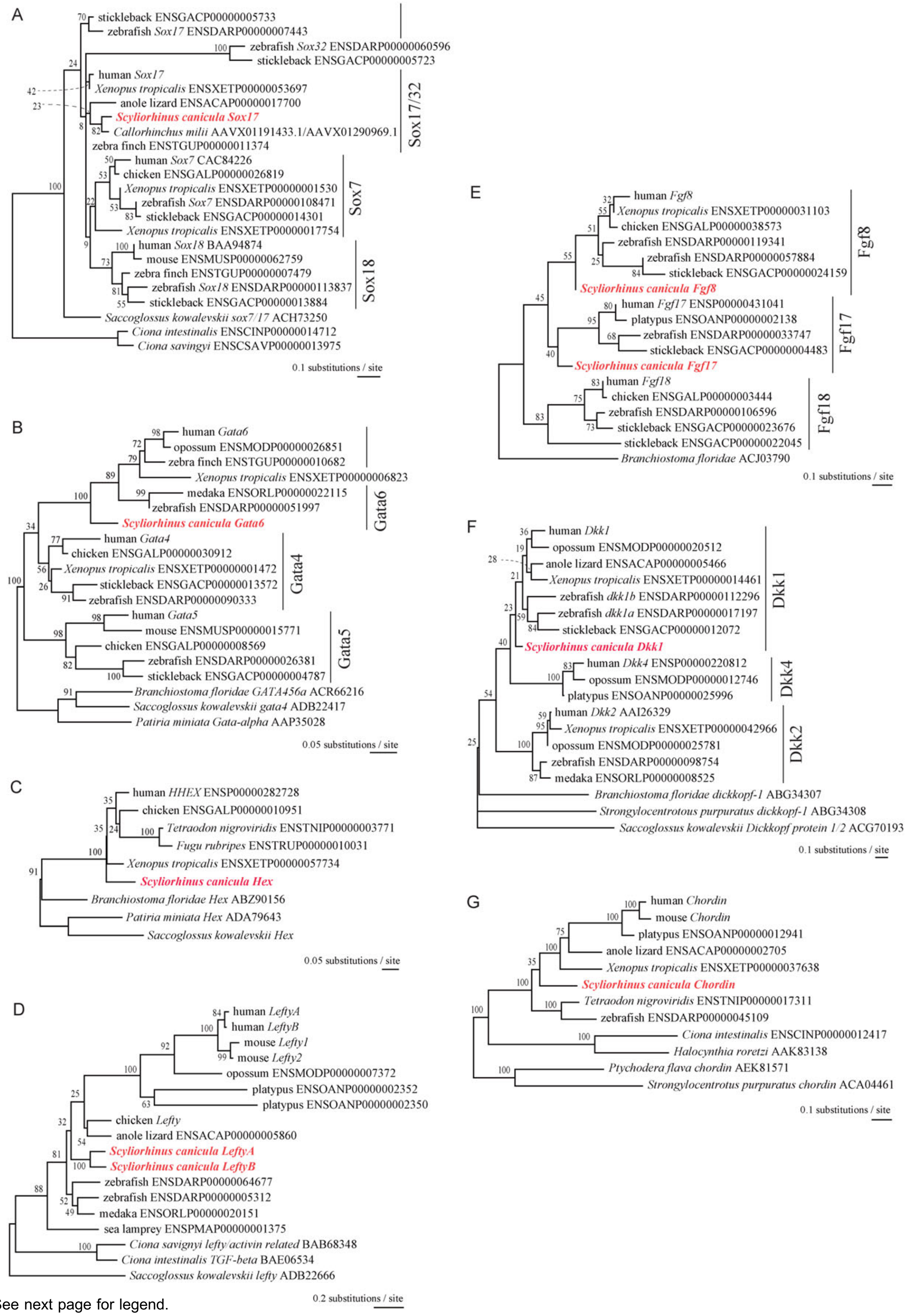

Fig. S1. See next page for legend.

Biology Open

Biology Open (2014) 000, 1–10 doi:10.1242/bio.20148037

Fig. S1. Molecular phylogeny of the *S. canicula* genes identified in this study. Maximum-likelihood (ML) trees were inferred using the program PhyML version 3.0 (Guindon and Gascuel, 2003) for *Sox7/17/18* (A), *Gata6* (B), *Hex* (C), *Lefty* (D), *Fgf8/17/18* (E), *Dkk1* (F) and *Chordin* (G). The sequence alignments were constructed with the alignment editor XCed in which MAFFT is implemented (Katoh et al., 2005) including the deduced amino acid sequences of the *S. canicula* cDNA identified in this study as well as peptide sequences of other species retrieved from the Ensembl and GenBank databases. The JTT model of amino acid substitutions was assumed with the among-site rate heterogeneity based on the gamma distribution taken into account. The shape parameter for the gamma distribution that maximizes the likelihood of a preliminary neighbor-joining tree was computed for each gene. The numbers of the amino acid residues in the input alignments were: 82 amino acids (aa) (A); 155 aa (B); 128 aa (C); 167 aa (D); 51 aa (E); 108 aa (F); 611 aa (G). Accession numbers for the catshark sequences are the following: *ScSox17*, KJ190304; *ScLeftyB*, KJ190305; *ScChd*, KJ190306; *ScShh*, KJ190307; *ScGata6*, KJ190308; *ScFgf17*, KJ190309; *ScHex*, KJ190310; *ScDkk1*, KJ190311.

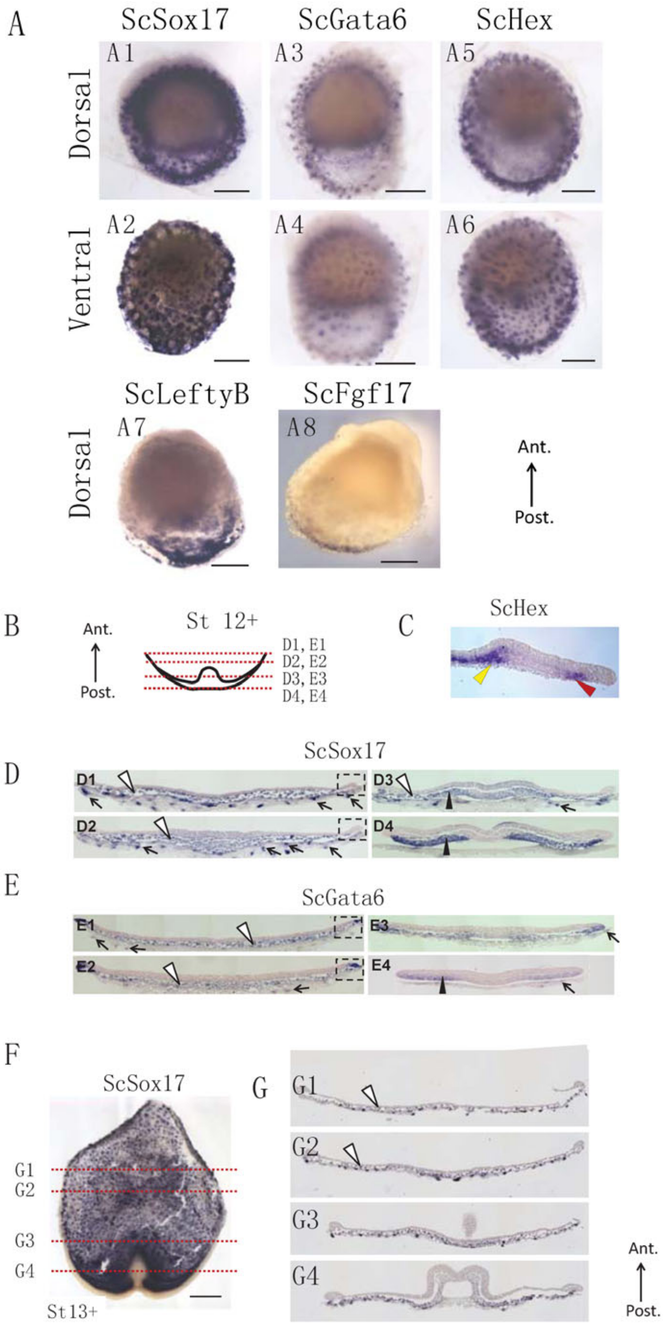

Fig. S2. Additional sections of embryos hybridized with *ScHex*, *ScSox17* and *ScGata6* probes. (A) Views of catshark embryos less than 48 hours after egg deposition (stages 4–6) after whole-mount in situ hybridizations with *ScSox17* (A1,A2), *ScGata6* (A3,A4), *ScHex* (A5,A6), *ScLeftyB* (A7) and *ScFgf17* (A8) probes. (A1,A3,A5,A7,A8) Dorsal views. (A2,A4,A6) Ventral views. (B) Scheme of the posterior part of a stage 12 catshark blastoderm, showing the level of the transverse sections shown in panels D and E. (C) Mid-sagittal section of a stage 12+ embryo following whole-mount hybridization with the *ScHex* probe. (D,E) Transverse sections following whole-mount hybridization with *ScSox17* and *ScGata6* probes respectively. (F,G) Ventral view (F) and transverse sections (G) of a stage 13+ catshark embryo following whole-mount in situ hybridization with a *ScSox17* probe. The red and yellow arrowheads in panel C show *ScHex* signals respectively in the presumptive prechordal mesendoderm derived from the early notochordal triangle and in the anterior-most region of the involuting mesendoderm where the foregut diverticulum forms. White arrowheads point to labeled cells in the deep mesenchyme, black arrowheads point to the labeled part of the involuting mesendoderm, thin arrows point to labeled yolk syncytial nuclei. Dotted boxes in the sections presented in panels D1, D2, E1 and E2 show the lateral blastoderm margins, where differences between *ScGata6* and *ScSox17* are observed. Red dotted lines indicate section planes and level. The antero-posterior axis is indicated at the right of the pictures. White arrowheads point to labeled deep mesenchymal cells, which persist at this stage. Scale bars: 500 µm.

Biology Open (2014) 000, 1–10 doi:10.1242/bio.20148037

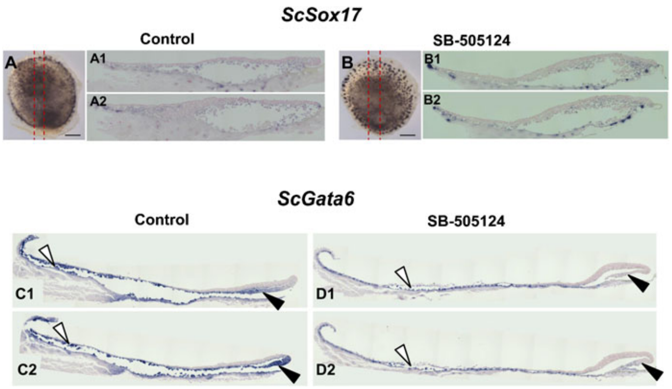

Fig. S3. Sagittal sections of control and SB-505124 type 1 and type 2 catshark embryos, following hybridizations with respectively *ScSox17* and *ScGata6* probes. (A,A1,A2,C1,C2) Control embryos, (B,B1,B2,D1,D2) SB-505124 treated embryos, (A1,B1,C1,D1) mid-sagittal sections, (A2,B2,C2,D2) para-sagittal sections (section levels indicated by dotted red lines for panels A1, A2, B1 and B2. The posterior overhang is either absent (Type 1 embryos, (B)) or present but less extended with a thinner IME (involuting mesendoderm) layer (Type 2 embryos, (D1,D2)) in SB-505124 embryos. Deep mesenchymal cells (white arrowheads) express *ScSox17* and *ScGata6* in SB-50124 treated embryos as in controls but the signal is lost in the IME layer (black arrowheads; magnifications of panels C1 and D1 at the level of the posterior margin are shown in Fig. 7M1′,N1′).

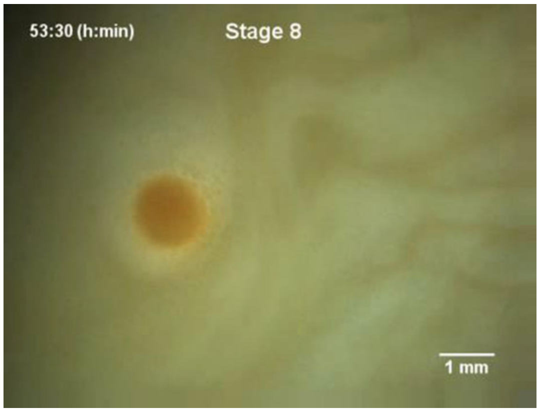

Movie 1. Time lapse imaging of a developing catshark embryo (stages 6 to 10). The movie shows a dorsal view of a catshark embryo from stages 6 to 10, anterior (referring to the orientation of the future embryonic axis) to the left. At early blastula stages (stages 6–7), the blastoderm is round-shaped, less than 1.5 mm diameter and surrounded by a characteristic white area. The blastocoel appears as a crescent located to the posterior side of the blastoderm at stage 6 and disappears at stage 7. Stages 8 to 9 (midblastula) are characterized by a size expansion and the partial (stage 8) to complete (stage 9) disappearance of the white area surrounding the blastoderm. Stage 10 is marked by the appearance of a thickening of the posterior side of the blastoderm. For the movie, a window was opened in the eggshell at the embryo level and photographs were taken every 15 minutes using an Olympus SZX12 stereomicroscope and a Qimaging colour 12-bit digital camera for the duration indicated in the movie.

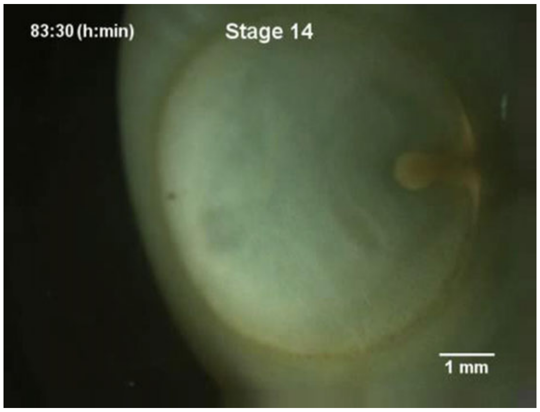

Movie 2. Time lapse imaging of a developing catshark embryo (stages 11 to 15). The movie shows a dorsal view of a catshark embryon from stages 11 to 15, anterior to the left. The embryonic axis proper forms in the posterior part of the blastoderm, while anterior and lateral blastoderm territories spread over the yolk to form the yolk sac. Stage 11 is characterized by the morphological appearance of the involuting mesendoderm layer, which extends over the yolk at the posterior margin. At stage 12, the *ScT* positive (Sauka-Spengler et al., 2003), bilaterally symmetrical extensions which form on each side of the midline at the posterior margin are referred to as the posterior arms and the midline, *ScChordin* positive groove visible at this level (this study) is referred to as the notochordal triangle, following the nomenclature of Ballard et al. (Ballard et al., 1993). The anterior-most part of the embryonic axis, positive for *ScGsc* and *ScOtx1/2*, forms at this stage (Coolen et al., 2007). At this stage, trunk markers are restricted to the posterior arms (Coolen et al., 2007). The neural groove becomes visible in the anterior part of the forming embryonic axis at stage 13 while mesendoderm internalization progresses at the trunk level, as assessed by *ScT* and trunk markers expression (Sauka-Spengler et al., 2003; Coolen et al., 2007). At subsequent stages, the embryonic axis elongates from anterior to posterior with a sharp temporal regulation (Coolen et al., 2007). At stage 14, the neural plate exhibits an enlargement in the cephalic region, and the neural folds lying between the prospective epidermis and the neural plate become clearly delineated. At stage 15, the neural groove hollows and the neural folds get closer together (Coolen et al., 2007). We refer to the mesenchymal cell population in contact with the yolk as deep mesenchyme (dm) and to the involuting lower cell layer observed starting from stage 11 (onset of gastrulation) as involuting mesendoderm (ime). For the movie, a window was opened in the eggshell at the embryo level and photographs were taken every 15 minutes using an Olympus SZX12 stereomicroscope and a Qimaging colour 12-bit digital camera for the duration indicated in the movie.

Biology Open (2014) 000, 1–10 doi:10.1242/bio.20148037

Table S1. Location of labeled cells following DiI application at the posterior margin of stage 9 to 11 catshark embryos

Culture time	1 h	24 h		
Injection stage	St. 9–11	St. 9	St. 10	St. 11
Resulting stage	St. 10–12	St. 10	St. 11	St. 12
Number of embryos analyzed	7	4	2	2
Number of embryos showing labeled cells in the location indicated	PM: 7/7	DM: 4/4<br>IME: 0/4	DM: 0/2<br>IME: 2/2	DM: 0/2<br>IME: 2/2

PM, posterior margin superficial cells; DM, deep mesenchyme; IME, involuting mesendoderm.

Table S2. Presence of labeled cells within the deep mesenchyme (DM) following DiI application in the center of the blastoderm

Culture time	1 h	24 h	
Injection stage	St. 9–10	St. 9–9+	St. 10–10+
Resulting stage	St. 10–11	St. 10–10+	St. 11–11+
Number of embryo analyzed	4	3	3
Number of embryos showing labeled cells in the DM	0/4	3/3	3/3

Table S3. Number of control and SB-505124 treated embryos analyzed

	Control	SB 505124	
	St. 11–12	Class 1	Class 2
LeftyB	3	3	0
Brachyury	5	4	0
Chordin	2	3	0
Otx5	4	2	0
Sox17	4	3	2
Gata6	5	2	3
Hex	2	3	0
Lim1	4	4	0

Biology Open
